# Epidemiology of *Blastocystis* sp. infection in China: a systematic review

**DOI:** 10.1051/parasite/2019042

**Published:** 2019-07-16

**Authors:** Lei Deng, Yijun Chai, Ziyao Zhou, Haifeng Liu, Zhijun Zhong, Yanchun Hu, Hualin Fu, Chanjuan Yue, Guangneng Peng

**Affiliations:** 1 The Key Laboratory of Animal Disease and Human Health of Sichuan Province, College of Veterinary Medicine, Sichuan Agricultural University Chengdu Sichuan 611130 PR China; 2 Sichuan Key Laboratory of Conservation Biology for Endangered Wildlife Chengdu Sichuan Province 611130 PR China

**Keywords:** *Blastocystis*, Molecular epidemiology, China, Zoonosis

## Abstract

*Blastocystis* sp., a unicellular intestinal parasite in humans and animals worldwide, is frequently found in immunocompromized patients and people in close contact with animals. Here, we reviewed recent studies on the prevalence, subtypes, and distribution of *Blastocystis* infection in humans and animals in China. To date, more than 12 provinces have reported *Blastocystis* infection in humans, with identification of six different subtypes (ST1, ST2, ST3, ST4, ST5, and ST6). The overall infection rate reported was 3.37% (3625/107,695), with the lowest prevalence (0.80%) in Fujian province and the highest prevalence (100%) in Guangdong province. ST3 (62%, 186/300) was the most dominant subtype, identified in all tested provinces in China. A total of eight provinces have reported *Blastocystis* infection in various animals, with the overall prevalence being 24.66% (1202/4874). Molecular analysis revealed 14 subtypes that infected animals, including 10 known (ST1, ST2, ST3, ST4, ST5, ST6, ST7, ST10, ST13, ST14), and 4 novel (Novel1, Novel2, Novel3, Novel4) subtypes. ST5 was the dominant subtype infecting artiodactyls (44.1%, 460/1044), while ST1 commonly infected carnivores (45.5%, 5/11). These findings provide insights into the epidemiological behavior of *Blastocystis* sp. in China, and could help in developing effective control strategies against the parasite.

## Abstract (fr)

*Blastocystis* sp., un parasite intestinal unicellulaire de l’homme et des animaux dans le monde entier, est fréquemment détecté chez les patients immunodéprimés et les personnes en contact étroit avec les animaux. Ici, nous avons passé en revue des études récentes sur la prévalence, les sous-types et la distribution de l’infection à *Blastocystis* chez l’homme et l’animal en Chine. À ce jour, plus de 12 provinces ont signalé une infection à *Blastocystis* chez l’homme, avec l’identification de 6 sous-types différents (ST1, ST2, ST3, ST4, ST5 et ST6). Le taux d’infection global signalé était de 3,37 % (3625/107695), la prévalence la plus faible (0,80 %) étant dans la province du Fujian et la prévalence la plus élevée (100 %) dans la province du Guangdong. ST3 (62 %, 186/300) était le sous-type le plus dominant, identifié dans toutes les provinces testées en Chine. Au total, 8 provinces ont signalé une infection à *Blastocystis* chez divers animaux, avec une prévalence globale de 24,66 % (1202/4874). L’analyse moléculaire a révélé 14 sous-types infectant des animaux, dont 10 sous-types connus (ST1, ST2, ST3, ST4, ST5, ST6, ST7, ST10, ST13, ST14) et 4 nouveaux (Novel1, Novel2, Novel3, Novel4). ST5 était le sous-type dominant infectant les artiodactyles (44,1 %, 460/1044), tandis que ST1 infectait couramment les carnivores (45,5 %, 5/11). Ces résultats fournissent des informations sur le comportement épidémiologique de *Blastocystis* sp. en Chine et pourrait aider à élaborer des stratégies de contrôle efficaces contre le parasite.

## Introduction

*Blastocystis* sp. is an anaerobic intestinal parasite infecting humans and several animals [51, 52]. *Blastocystis* sp. was previously thought to be a fungus (non-pathogenic yeast), but was later identified as belonging to the Stramenopiles, a complex and heterogeneous evolutionary assemblage of heterotrophic and photosynthetic protozoa [38, 43]. The transmission of *Blastocystis* sp. can be direct or indirect between individuals via the fecal-oral route, which is similar to that observed with some intestinal protozoans, including *Cryptosporidium* spp., *Entamoeba* spp., *Giardia intestinalis*, and *Dientamoeba fragilis* [49, 63]. Several studies have shown that contaminated water and food with a few number of *Blastocystis* cysts can establish an infection [17, 62]. Clinical presentations caused by this parasite are very diverse, ranging from self-limiting abdominal discomfort to chronic persistent diarrhea [33]. Frequently, these parasitic infections also present with dermatological symptoms, depending on the different *Blastocystis* subtypes [14]. Compared to healthy individuals, a higher incidence of *Blastocystis* has been observed in patients with diarrhea or other gastrointestinal symptoms, especially in patients with irritable bowel syndrome (IBS) symptoms [5, 18]. Since its first descriptions by Alexeieff and Brumpt [12], *Blastocystis* sp. has been found in a wide host range of animal hosts, including artiodactyls, perissodactyls, proboscideans, rodents and marsupials as well as birds, reptiles, amphibians, fish, annelids, and insects [31, 48]. *Blastocystis* sp. is included in the Water, Sanitation, and Health programs of the World Health Organization [58].

Polymerase chain reaction (PCR)-based approaches using feces directly or after culture of fecal specimens have been developed for the diagnosis of *Blastocystis* infection [27, 39]. More recently, Poirier et al. reported a highly sensitive real-time quantitative PCR (qPCR) assay that targeted a region of the small subunit rRNA gene (SSU-rDNA) and allowed subtyping of isolates in stool samples by direct sequencing of the qPCR products [36]. Moreover, a qPCR assay using the SSU-rDNA marker was also developed, including an internal process control enabling the evaluation of potential PCR inhibitors [8]. Therefore, SSU-rDNA genotyping is the method of choice for diagnosis [8]. Based on SSU-rDNA genotyping, high genetic variability was observed for *Blastocystis* sp., and 17 known subtypes (ST1-ST17) have been reported [2, 29, 40, 41]. Of these, subtypes 1–9 have been found in humans, with ST1–ST4 as the prevalent subtypes in humans that were identified in >90% of investigations [21]. Some human subtypes were also observed in animals, e.g., ST3 in non-human primates, ST5 in cattle and pigs, and ST7 in birds [32]. Additionally, ST5 was commonly detected in pigs and their in-contact handlers (piggery staff) in Australia, indicating the zoonotic potential of this subtype [61].

Owing to its important role in clinical and public health, *Blastocystis* has attracted the attention of many Chinese scientists, who have made significant contributions to the characterization of *Blastocystis* sp. and understanding of their biology and transmission. Since the first report of *Blastocystis* sp. in humans in China in 1996 [24], high prevalence and abundant genetic diversity of *Blastocystis* sp. have been observed in humans, non-human primates (NHPs), domestic animals (e.g., cattle, sheep, goats, and pigs), and wild animals from many provinces of China. Herein, we review the current knowledge on the epidemiology and subtyping of *Blastocystis* sp. in humans and animals in China.

## Characteristics and distribution of *Blastocystis* sp. in humans

**Figure 1:**
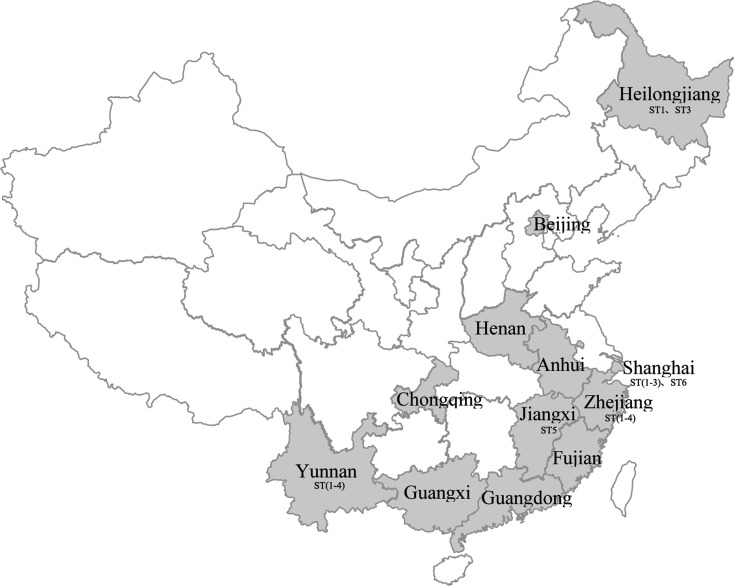
Prevalence of human *Blastocystis* in different provinces/municipalities in China.

Human *Blastocystis* sp. infection has been widely reported in the world, in developing countries (Iran, Jordan, Argentina, Egypt, Thailand, Philippines, Malaysia, Zambia, Indonesia, Chile, and China) as well as developed countries (Japan, Singapore, England, Spain, Italy, Germany, United States, and Turkey) [30, 42, 46]. The first description of *Blastocystis* sp. infection in China was reported in two children with chronic diarrhea from Guangdong province [24]. Subsequently, more than 12 provinces/municipalities have reported *Blastocystis* sp. infection in China (Fig 1). Most of these reports investigated *Blastocystis* sp. infection in patients with diarrhea [19, 50], and the prevalence ranged from 0.80% to 100% (Table 1). The average prevalence was 3.37% (3625/107,695), with the highest infection rate being 32.6%, (78/239) reported using PCR amplification of the SSU-rDNA gene of *Blastocystis* sp. in Yunnan province [22], and the lowest rate being 0.80%, (85/10,652) determined by a method using direct saline smear and iodine staining in Fujian province [7].

**Table 1:** Prevalence of *Blastocystis* sp. in humans in China.

Location	Method of diagnosis	No. examined	No. positive	Prevalence (%)	Diarrhea	Subtypes (*n*)	References
Guangdong	*In vitro* culture	2	2	100%	Yes		[24]
Jiangxi	*In vitro* culture	46,900	1122	2.39%	Yes		[59]
	PCR	3	3	100%	NG	ST5 (2), ST3 + ST5 (1)	[61]
Subtotal		469,903	1125	2.40%		ST5 (2), ST3 + ST5 (1)	
Anhui	Smear/Iodine/Hematoxylin	703	26	3.70%	Yes		[57]
Chongqing	Smear	2558	35	1.37%	NG		[54]
Guangxi	Smear/Trichrome	1354	251	18.54%	Yes		[19]
	Smear	39,671	1551	3.91%	Yes		[15]
	Smear/Iodine	1185	145	12.24%	Yes		[16]
Subtotal		42,210	1947	4.61%			
Yunnan	PCR	239	78	32.64%	NG	ST3 (56), ST1 (16), ST2 (1), ST4 (1), ST1 and 2 (1), ST1 and 3 (1), Unknown (3)	[21]
	PCR	170	10	5.88%	NG	ST3 (6), ST1 (3), ST2 (1)	[22]
	PCR	1020	37	3.63%	Yes		[47]
	Smear	215	43	20.0%	Yes		[65]
Subtotal		1644	168	10.22%		ST3 (62), ST1 (19), ST2 (2), ST4 (1), ST1 and 2 (1), ST1 and 3 (1), Unknown (3)	
Shanghai	PCR	1505	29	1.90%	NG	ST3 (17), ST1 (6), ST2 (1), ST6 (1), ST1 and 3 (2), Unknown (2)	[21]
Zhejiang	PCR	646	153	23.68%	NG	ST3 (93), ST1 (38), ST2 (7), ST4 (1), ST1 and 3 (6), ST1 and 2 (1), ST2 and 3 (1), Unknown (6)	[21]
Beijing	Smear/Trichrome	122	6	4.92%	Yes		[50]
Henan	Smear/Iodine/*In vitro* culture	369	22	5.96%	Yes		[25]
Fujian	Smear/Iodine	10,652	85	0.80%	NG		[7]
Heilongjiang	PCR	381	27	7.10%	Yes	ST1 (12), ST3 (15)	[55]
Total		107,695	3625	3.37%		ST3 (186), ST1 (75), ST2 (10), ST4 (2), ST5 (2), ST6 (1), ST1 and 3 (9), ST1 and 2 (2), ST2 and 3 (1), ST3 + ST5 (1), Unknown (11)	

NG: Not given.

The prevalence of *Blastocystis* sp. infection may be affected by many factors, such as the immune status of hosts, different geographic locations, age of hosts, and eating habits. For example, the prevalence was higher in patients with different degrees of diarrhea than in those without gastrointestinal illnesses [57, 65]. The prevalence was also higher in rural communities than in urban residents [7, 54], and higher prevalence was observed in the southern regions with less developed inland areas compared with that in the northern coastal provinces [21, 47]. Further, the infection rate was higher in people aged from 18 to 39 years than in children <5 years of age in Yunnan province [22], which was consistent with the observations in Guangxi [15] and Heilongjiang provinces [66]. In addition, infection with *Blastocystis* sp. was associated with drinking un-boiled water in a hilly village in the Yunnan province of southern China [22]. In 2000, there was an outbreak of *Blastocystis* sp. infection, in which humans were infected through ingestion of contaminated running water, and more than 1122 patients with diarrhea were identified [59]. These results suggest that *Blastocystis* sp. might be transmitted by contaminated water to humans in China.

Sequence analysis of the barcode region of the SSU rRNA gene in isolates identified a total of six different *Blastocystis* sp. subtypes in China (Table 1). Most of the samples represented single-subtype infections (ST1, ST2, ST3, ST4, ST5, and ST6), while mixed infections were also observed (ST1 + 2, ST1 + 3, ST2 + 3, ST3 + 5), and some subtypes were novel (unknown). Among them, ST3 (62%, 186/300) was the most dominant subtype of *Blastocystis* sp., and was identified in almost all studies reported in China based on PCR analysis. A large majority of human infections were also attributable to the ST3 isolates reported in Europe, Africa, Oceania, and the Middle East [13, 32, 43, 48].

## Characteristics and distribution of *Blastocystis* sp. in animals

**Figure 2:**
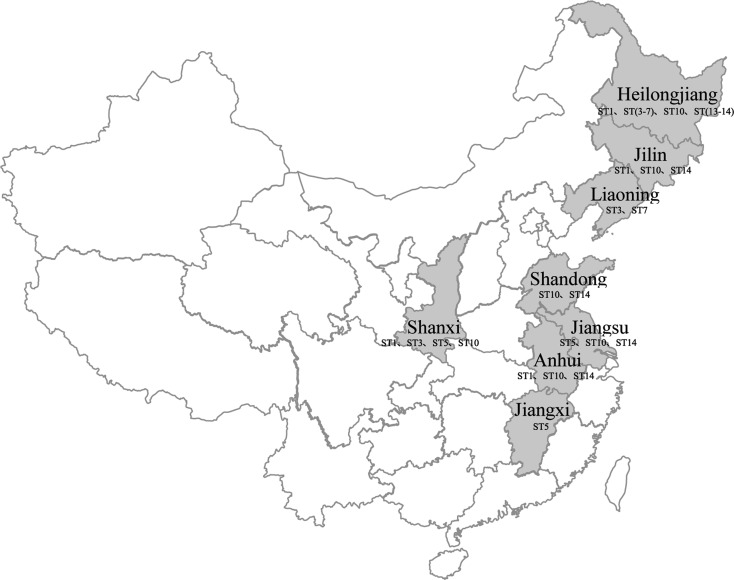
Prevalence of animal *Blastocystis* in different provinces/municipalities in China.

*Blastocystis* sp. has been isolated from a variety of animals worldwide, including in Spain, the UK, Czechoslovakia, Japan, Thailand, Malaysia, Ethiopia, Indonesia, Chile, Australia, Singapore, and China [30]. The prevalence varies from 1.8% identified in cattle in Spain to 100% identified in chickens in Japan [28, 37]. In China, *Blastocystis* infection has been reported in 25 different species among the orders Artiodactyla, Carnivora, Galliformes, Primates, Columbiformes, Anseriformes, Gruiformes, Rodentia, and Lagomorpha from eight different provinces (Fig. 2). The average infection rate was 24.66% (1202/4874), with the prevalence of infection varying markedly among different orders and different geographical areas in China (Table 2).

**Table 2:** Prevalence of *Blastocystis* sp. in animals in China.

Order	Location	Method of diagnosis	Hosts (Scientific name)	No. examined	No. positive	Prevalence (%)	Subtypes (*n*)	References
Artiodactyla	Shanxi	Smear/Iodine	Wild boars (*Sus scrofa*)	5	1	20.0%		[6]
	Shanxi	Smear/Iodine	Himalayan Goral (*Naemorhedus goral*)	11	2	18.20%		[6]
	Shanxi	PCR	Pig	560	419	74.82%	ST5 (397), ST1 (15), ST3 (6), ST10 (1)	[44]
	Jiangxi	PCR	Pig	16	16	100%	ST5 (16)	[61]
	Heilongjiang	PCR	Pig	68	6	8.82%	ST5 (6)	[55]
	Heilongjiang	PCR	Cattle	147	14	9.52%	ST10 (10), ST3 (2), ST14 (2)	[55]
	Heilongjiang	PCR	Dairy cattle	526	54	10.27%	ST10 (41), ST14 (10), ST4 (2), ST5 (1)	[45]
	Heilongjiang	PCR	Sheep	109	6	5.50%	ST10 (3), ST1 (1), ST5 (1), ST14 (1)	[55]
	Anhui	PCR	Sheep	697	22	3.16%	ST10 (14), ST14 (3), Novel 1 (3), Novel 2 (1), Novel 3 (1)	[23]
	Jiangsu	PCR	Sheep	75	18	24.0%	ST5 (8), ST10 (5), ST14 (5)	[23]
	Shandong	PCR	Sheep	60	10	16.67%	ST10 (6), ST14 (2), Novel 4 (2)	[23]
	Shanxi	PCR	Goat	789	458	58.05%	ST10 (292), ST14 (123), ST5 (31), ST4 (9), ST1 (1), ST3 (1), Novel (1)	[43]
	Anhui	PCR	Goat	574	2	0.35%	ST1 (2)	[43]
	Heilongjiang	PCR	Reindeer (*Rangifer tarandus*)	104	7	6.73%	ST13 (4), ST10 (3)	[56]
	Heilongjiang	PCR	Sika deer (*Cervus nippon*)	52	6	11.54%	ST10 (6)	[56]
	Jilin	PCR	Sika deer (*Cervus nippon*)	30	6	20%	ST10 (4), ST14 (2)	[56]
	Shanxi	Smear/Iodine	Barking Deer (*Muntiacus muntjak*)	2	2	100%		[6]
	Shanxi	Smear/Iodine	Sika deer (*Cervus nippon*)	3	1	33.33%		[6]
Subtotal				3828	1050	27.43%	ST5 (460), ST10 (385), ST14 (148), ST1 (19), ST4 (11), ST3 (9), ST13 (4), Novel1 (4), Novel2 (1), Novel3 (1), Novel4 (2)	
Carnivora	Heilongjiang	PCR	Racoon dog (*Nyctereutes procyonoides*)	16	1	6.25%	ST3 (1)	[56]
	Liaoning	PCR	Racoon dog (*Nyctereutes procyonoides*)	24	2	8.33%	ST3 (2)	[56]
	Heilongjiang	PCR	Domestic dog	124	2	1.61%	ST1 (1), ST4 (1)	[56]
	Jilin	PCR	Domestic dog	12	2	16.67%	ST1 (2)	[56]
	Liaoning	PCR	Arctic fox (*Vulpes lagopus*)	40	1	2.5%	ST7 (1)	[56]
	Heilongjiang	PCR	Arctic fox (*Vulpes lagopus*)	173	3	1.73%	ST1 (2), ST4 (1)	[56]
Subtotal				389	11	2.83%	ST1 (5), ST3 (3), ST4 (2), ST7 (1)	
Galliformes	Shanxi	Smear/Iodine	Peafowl (*Pavonini*)	9	3	33.33%		[6]
	Shanxi	Smear/Iodine	Brown-eared pheasant (*Crossoptilon mantchuricum*)	2	2	100%		[6]
	Shanxi	Smear/Iodine	Crimson-bellied tragopan (*Tragopan temminckii*)	2	1	50%		[6]
	Shanxi	Smear/Iodine	Golden pheasant (*Chrysolophus pictus*)	11	10	90.91%		[6]
	Heilongjiang	PCR	Domestic chicken	46	6	13.04%	ST6 (3), ST7 (3)	[56]
Subtotal				70	22	31.43%	ST6 (3), ST7 (3)	
Primates	Shanxi	Smear/Iodine	Golden monkey (*Rhinopithecus*)	12	8	66.67%		[6]
	China^a^	PCR	Cynomolgus macaques (*Macaca fascicularis*)	97	85	87.63%	ST1 (4), ST2 (14), ST7 (2), ST2 + ST1 (14), ST2 + ST3 (5), ST2 + ST7 (5), ST3 + ST1 (3), ST5 + ST2 (1), ST7 + ST1 (7), ST7 + ST3 (1), ST1 + ST2 + ST3 (10), ST1 + ST2 + ST7 (5), ST2 + ST3 + ST7 (3), ST1 + ST3 + ST7 (1), ST1 + ST2 + ST3 + ST7 (10)	[64]
Subtotal				109	93	85.32%	ST1 (4), ST2 (14), ST7 (2), ST2 + ST1 (14), ST2 + ST3 (5), ST2 + ST7 (5), ST3 + ST1 (3), ST5 + ST2 (1), ST7 + ST1 (7), ST7 + ST3 (1), ST1 + ST2 + ST3 (10), ST1 + ST2 + ST7 (5), ST2 + ST3 + ST7 (3), ST1 + ST3 + ST7 (1), ST1 + ST2 + ST3 + ST7 (10)	
Columbiformes	Heilongjiang	PCR	Pigeon	47	1	2.13%	ST6 (1)	[56]
	Shanxi	Smear/Iodine	Crested ibis (*Nipponia nippon*)	63	6	9.53%		
Subtotal				110	7	6.36%	ST6 (1)	
Anseriformes	Shanxi	Smear/Iodine	Whooper swan (*Cygnus cygnus*)	2	2	100%		[6]
Gruiformes	Heilongjiang	PCR	Red crowned crane (*Grus japonensis*)	43	6	13.95%	ST6 (4), ST7 (2)	[56]
Rodentia	Heilongjiang	PCR	Brown rat (*Rattus norvegicus*)	108	4	3.70%	ST4 (4)	[56]
Lagomorpha	Heilongjiang	PCR	New Zealand white rabbit	215	7	3.24%	ST4 (7)	[56]
Total				4874	1202	24.66%	ST5 (460), ST10 (385), ST14 (148), ST1 (28), ST4 (24), ST2 (14), ST3 (12), ST6 (8), ST7 (8), ST13 (4), ST2 + ST1 (14), ST2 + ST3 (5), ST2 + ST7 (5), ST3 + ST1 (3), ST5 + ST2 (1), ST7 + ST1 (7), ST7 + ST3 (1), ST1 + ST2 + ST3 (10), ST1 + ST2 + ST7 (5), ST2 + ST3 + ST7 (3), ST1 + ST3 + ST7 (1), ST1 + ST2 + ST3 + ST7 (10), Novel (4), Novel4 (2), Novel2 (1), Novel3 (1)	

a fecal samples from registered breeding facilities in China (F2 purpose-bred).

In Artiodactyla, pigs, cattle, sheep, goats, and deer are the commonly infected animals, with the prevalence ranging from 0.35% to 100%. Infection in pigs and cattle is also the most common in other countries, such as in Japan [1] (70.9% in cattle and 95.1% in pigs), and Spain (1.8% in cattle and 46.8% in pigs) [34]. The first report of *Blastocystis* sp. infection in sheep was from Heilongjiang province in 2018 [55]. Li et al. found that the highest infection rate of *Blastocystis* sp. in sheep was 16.67% [23], which was lower than that in sheep in the UK (23.5%) [2, 3] and Malaysia (30.9%) [53]. Variable prevalence was observed in different species of deer, ranging from 6.73% to 100% (Table 2). Molecular characterization of these *Blastocystis* sp. isolates from Artiodactyla indicated 11 subtypes, including 7 known subtypes (ST1, ST3, ST4, ST5, ST10, ST13, ST14) and 4 novel subtypes (Novel1, Novel2, Novel3, Novel4). Among these subtypes, ST5 was the most prevalent, accounting for 44.1% (460/1044). Interestingly, ST5 has been identified in humans and pigs in the rural areas of Jiangxi province, where many households keep small pigs, with pigs and children sometimes sharing common outdoor areas [61]. These reports suggest that ST5 shows zoonotic potential and pigs may be a potential source of infection in these rural areas.

In Carnivora, *Blastocystis* sp. infection has been commonly found in raccoon dogs (6.25–8.33%), domestic dogs (1.61–16.7%), and arctic foxes (1.73–2.5%). A total of four subtypes of *Blastocystis* sp. were identified (ST1, ST3, ST4, ST7), with ST1 being the most prevalent subtype (Table 2). Importantly, ST1 has been identified in humans in the same region, indicating that ST1 might have the ability to transmit between humans and dogs [66]. Similarly, ST1 has been identified in dogs and their owners in Australia [33], the Philippines, and Turkey [4], suggesting that dogs could be involved in the transmission of *Blastocystis* to humans.

In Galliformes, the peafowl (33.3%), brown-eared pheasant (100%), crimson-bellied tragopan (50%), golden pheasant (90.9), and domestic chicken (13%) have presented with *Blastocystis* infection. Among these, the prevalence of infection among chickens is consistent with that reported in Australia (74.4%) [20] and Japan (100%) [60]. Pigeon and crested ibis, belonging to Columbiformes, show prevalence of *Blastocystis* sp. of 2.13% and 9.53%, respectively. In both Anseriformes and Gruiformes, only one species has been identified with *Blastocystis* infection at present, respectively (Table 2). ST6 and ST7, identified in these orders, are the most common subtypes in birds and are generally considered to be avian subtypes [9]. In addition to birds, the two subtypes are occasionally found in some mammals: ST6 in pigs, cattle, goats, and dogs [55, 56]; ST7 in pigs, goats, cynomolgus monkeys, ruffed lemurs, and dogs [64]. In humans, ST6 and ST7 only constitute a small share (approximately 9%) of cases of blastocystosis [2, 3].

In primates, golden monkeys and cynomolgus macaques have been identified with *Blastocystis* sp. infection, the average prevalence being 85.32%, which was higher than that observed in primates in Australia (2.1%) [35], Malaysia (53.8%) [26], and Spain (66.6%) [11]. ST2 was the most prevalent subtype, and mixed infection was observed in this order (Table 2). Brown rat and New Zealand white rabbit, belonging to Rodentia and Lagomorpha respectively, show similar prevalences of infection (3.70% and 3.24%, respectively). ST4 is the most common in rodents and is also one of the four most common subtypes in humans (ST1–ST4) [10].

**Figure 3:**
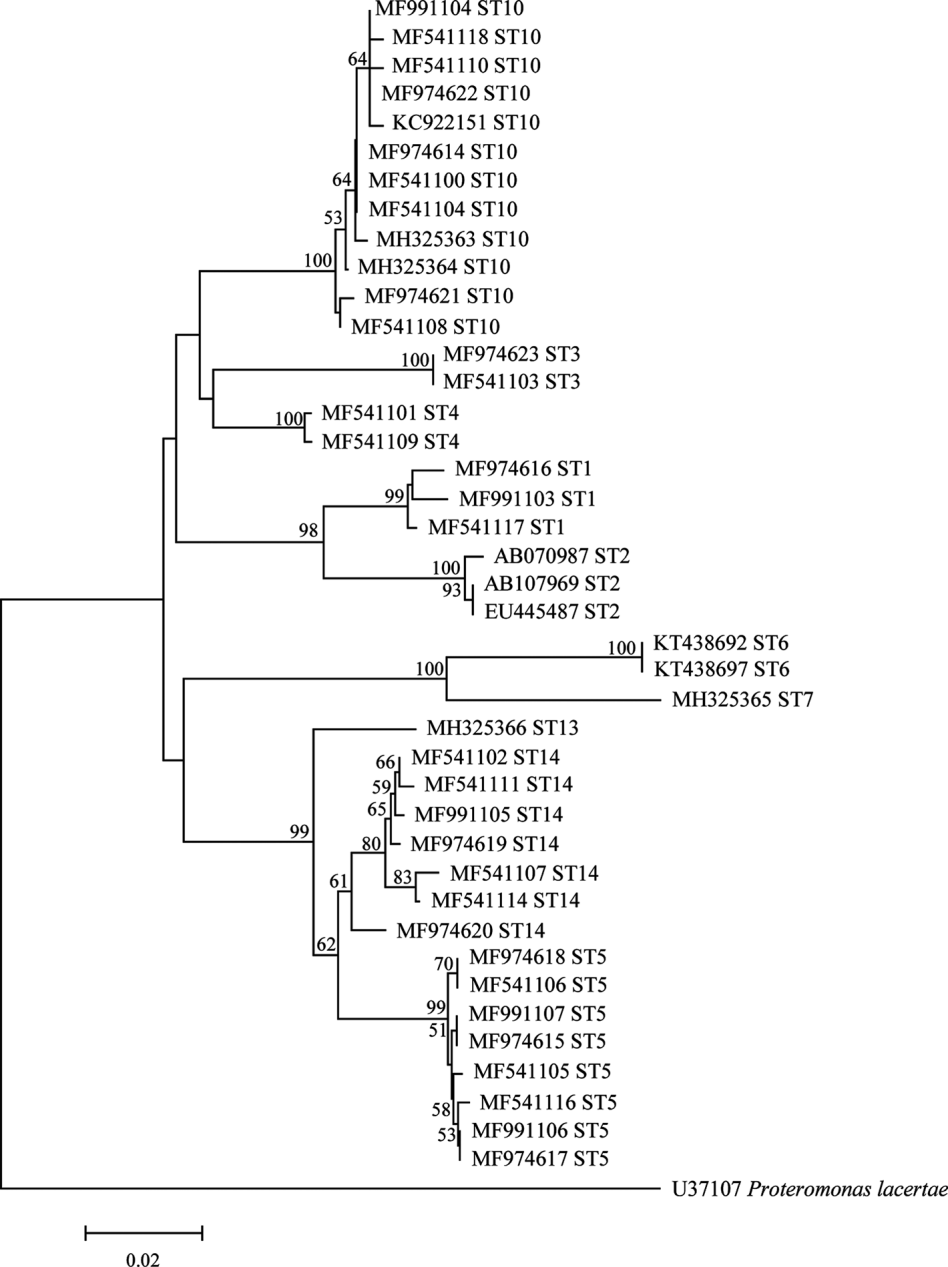
Phylogenetic relationships among the representative sequences of the *Blastocystis* small subunit ribosomal RNA (SSU rRNA) gene obtained from China, using the neighbor-joining method. The trees were rooted with GenBank sequence U37107. Bootstrap values greater than 50% from 1000 pseudoreplicates are shown.

Phylogenetic relationships were analyzed by the neighbor-joining method under the Kimura 2-parameter model using Mega 6 (http://www.megasoftware.net/), and a bootstrap analysis with 1000 replicates was performed to assess the robustness of clusters (Figure 3).

## Conclusions and perspectives

Since its first discovery in China in 1996, knowledge of the epidemiology of *Blastocystis* sp. has progressed significantly, with more than 12 provinces/municipalities having reported *Blastocystis* sp. infection in humans, and in over 25 different animal hosts. A total of 6 and 14 different subtypes of *Blastocystis* sp. have been identified in humans and animals, respectively. The most dominant subtype identified is ST3 in humans and ST5 in animals. Some subtypes (ST1, ST3, and ST5) have been found in humans and animals in the same province, suggesting that these zoonotic subtypes can be transmitted between humans and animals.

Additionally, although there is some evidence of fecal-oral transmission of the cyst form of *Blastocystis*, the actual mode of transmission among the various hosts and/or transmission between animals and humans has not yet been conclusively demonstrated. There is increasing evidence of the zoonotic potential of this parasite, although correlations between potential zoonotic subtypes and pathogenicity are still under debate. Therefore, more studies should be conducted to evaluate the pathogenicity, route of transmission, and host specificity of different *Blastocystis* sp. subtypes.
